# In Vitro Bioaccessibility and Health Risk Assessment of Arsenic and Zinc Contaminated Soil Stabilized by Ferrous Sulfate: Effect of Different Dietary Components

**DOI:** 10.3390/toxics11010023

**Published:** 2022-12-26

**Authors:** Yi Fang, Yuxue Cui, Xiaoli Mou, Li Lu, Jiali Shentu, Min Zhu

**Affiliations:** 1 Zhejiang Provincial Key Laboratory of Solid Waste Treatment and Recycling, Zhejiang Engineering Research Center of Non-Ferrous Metal Waste Recycling, School of Environmental Science and Engineering, Zhejiang Gongshang University, Hangzhou 310012, China; 2 Instrumental Analysis Center of Zhejiang Gongshang University, Hangzhou 310012, China

**Keywords:** contaminated site, heavy metal, stabilization, dietary, bioaccessibility, health risk

## Abstract

Iron-based materials have good stability in reducing the mobility and toxicity of heavy metals, but the behavior and human health risks of heavy metals could be affected by dietary components. This study investigated the effect of typical diets (lettuce, cooked rice and apples) on the bioaccessibility and morphological changes of arsenic (As) and zinc (Zn) in contaminated site after stabilization by ferrous sulfate (FeSO_4_). The results showed that the bioaccessibility of As and Zn were increased in a co-digestion system of food. The augmented effect on As bioaccessibility mainly occurred in the gastric phase: apple > lettuce > cooked rice (*p* < 0.05), while the augmented effect on Zn bioaccessibility mainly occurred in the intestinal phase: lettuce > apple > cooked rice (*p* < 0.05). FeSO_4_ weakened the dissolution effect of dietary components on As bioaccessibility, and reduced As bioaccessibility in the gastric and intestinal phases by 34.0% and 37.9% (*p* < 0.05), respectively. Dietary components and Fe fractions influenced the speciation and distribution of As and Zn. FeSO_4_ reduced the hazard quotient (HQ) and carcinogenic risk (CR) values of the contaminated soil by 33.97% and 33.59%, respectively. This study provides a reference for a better understanding of more realistic strategies to modulate exposure risks of heavy metal-contaminated sites.

## 1. Introduction

Arsenic (As) is a geogenic and anthropogenic released contaminant that seriously threatens human health and environmental safety, and environmental behavior/pollution control of soil As has always been the focus of research in the world [1,2,3]. It was reported that nearly 20 million people in China live in areas at high risk of As contaminated soil caused by industrial activities, such as smelting and plating [4,5,6]. Smelting wastes, tailings and combustion residues were discarded near the smelting plant, resulting a large number of harmful metals penetrated into the soil and destroyed the ecological structure of soil directly [4,7]. It reported that most of the soil As around a lead (Pb)/zinc (Zn) smelter exceeded the risk screening values in “Risk control standard for soil contamination of development land of China” (GB 15618–2018) (20 mg·kg^−1^) [5]. As and Zn had different behaviors and transport mechanisms in complex soil environments because As existed in the form of oxygen-containing anions, while Zn existed in the form of cations [8]. Chemical stabilization is a common risk controlling technology for heavy metals contaminated soils [9]. Stabilizers could change the form of heavy metals into less toxic forms to prevent the leaching of harmful substances from contaminated soil [10]. Iron-based materials had good stability in reducing the mobility and toxicity of metals due to good adsorption properties [11]. Ferrous sulfate (FeSO_4_) could react with arsenate to form the stable final products, and the resulting ferric hydroxide or magnetite could provide rich active sites to capture arsenic [12]. Studies have shown that the leaching concentration of As in soil reduced from 2.1 mg·L^−1^ to 0.2 mg·L^−1^ with 4.0 wt% FeSO_4_·7H_2_O [13]. The pilot test also verified that the stabilization of realgar tailings achieved by adding FeSO_4_, and the leaching amount of As decreased significantly with the increase of dosage [14,15].

Oral intake is the main exposure way of heavy metals from soil to human beings. It is important to determine whether the metals absorbed by gastrointestinal tract be decreased by FeSO_4_. The physiology-based extraction test (PBET) is widely used to assess the bioaccessibility of metals in the gastrointestinal tract due to its strong correlation with in vivo animal models [16]. It shown that the FeSO_4_ and phosphate effectively reduced soil Pb bioaccessibility in vivo and in vitro [17]. Hybrid iron-, sulfate- and phosphate-based bio-nanocomposites could decrease soil As bioaccessibility in gastric phase from 64.36% to 51.24% [18], and the toxicity of metals ingested could be reduced by dietary regulation [19]. Spinach, cola or vitamin C could reduce the bioaccessibility of Pb in the gastrointestinal stage or reduced the blood Pb level [20]. Vegetables and other edible plants containing large amounts of dietary fiber, minerals, carotenoids and sulfur-containing amino acids affected metal bioaccessibility [21]. Cooked rice, vegetables and fruits are the main types of Chinese daily diet, but their effects on heavy metal bioaccessibility in soil are still unclear. 

This study was conducted to evaluate the effect of cooked rice, vegetable and fruit on soil heavy metal bioaccessibility stabilized by FeSO_4_. The main purposes were: (1) to determine the effects of dietary components on As and Zn bioaccessibility in human gastrointestinal tract before and after stabilization; (2) revealing the mechanism of stabilization by determining the distribution and morphological changes of As, Zn, Fe in stomach and intestine after food intake; and (3) accurately assess the health risk of heavy metal contaminated soil after stabilization ingested by mouth, providing a scientific theoretical basis for nutrition management.

## 2. Materials and Methods

### 2.1. Collection and Pretreatment of Soil and Food Samples

Soil samples were collected from an iron and steel plant contaminated site in Zhejiang Province, eastern of China, which was established in 1957. Soil total Fe content was as high as 215.9 g∙kg^−1^, and it was also polluted by heavy metals, such as As, Zn and Pb. The original soil (HG) was taken from the soil surface layer (0~50 cm). 2% *w*/*w* FeSO_4_ was added into the soil. All treated soil was divided into two parts. A part of the soil was mixed evenly to evaluate the physicochemical properties after air drying, grinding and sieving through a 2 mm nylon sieve, the other part of the soil was sieved through a 0.149 mm nylon sieve and homogeneously mixed for in vitro experiment. All soil samples were stored in polyethylene bags at room temperature (25 °C) for the subsequent experimental analysis. The lettuce and apple were purchased from a vegetable market and washed with deionized water. Cooked rice was purchased from the canteen of Zhejiang Gongshang University. All food samples collected were dried to a constant weight at 60 °C and crushed with a pulverizer and sieved with a 0.149 mm nylon sieve. The samples were packed in bags and sealed for storage in a dryer for further use. 

### 2.2. Soil Properties and Heavy Metal Fractionations

The soil pH value was tested using a pH meter at 1:2.5 (*w*/*v*) ratio. Total organic carbon (TOC), dissolved organic carbon (DOC), total phosphorus (TP), total nitrogen (TN) and total potassium (TK) were measured according to previous studies [5]. To obtain the total concentration of heavy metals, 0.2 g of the soil sample was digested on an electric heating plate with HCl-HNO_3_-HF-HCIO_4_ solution. Soil available Zn and Fe were extracted by diethylenetriaminepentaacetic acid (DTPA), and soil available As was extracted by NH_4_H_2_PO_4_. The contents of Zn, Fe and As were determined by atomic absorption (iCE300) and atomic fluorescence spectrometer (AFS-9710), respectively. Sequential extraction procedure proposed by the European Community Bureau of Reference (BCR) was used to assess the fraction of Zn and Fe (ZF1/FF1: acid-exchangeable, ZF2/FF2: reducible, ZF3/FF3: oxidizable, ZF4/FF4: residual) in solid residues after PBET [22]. Wenzel sequential extraction method was used to assess the fraction of As (AF1: non–specifically bound As, AF2: specifically bound As, AF3: amorphous hydrous oxide bound As, AF4: crystalline hydrous oxide bound As, AF5: residual As) in solid residues after PBET [23].

### 2.3. In Vitro Bioaccessibility of Heavy Metals

The effect of dietary on soil heavy metal bioaccessibility was assessed at two stages according to PBET method. 1.25 g of pepsin, 0.5 g of sodium citrate, 0.5 g of sodium malate, 420 μL of lactic acid and 500 μL of glacial acetic acid were dissolved in 1 L of deionized water. The pH of the solution was adjusted to 2.00 ± 0.05, which served as a simulated gastric fluid. 0.3 g of soil sample and 30 mL (soil/liquid = 1:100) of gastric juice were placed in a polypropylene tube. Then, food was added to the gastric juice at different levels (0%, 25%, 50%, 100%, 200% and 400%, food/soil, *w*/*w*). All solutions were shaken in a constant temperature shaker at 100 rpm and 37 °C for 1 h. The supernatant was filtered through 0.22 μm membrane filter. The remaining solid residue was vacuum dried and used for heavy metal speciation analysis and mineral species detection. In order to simulate the transport of food from the stomach to intestine, the above simulated gastric juice was adjusted to pH 7.0, 0.5 g·L^−1^ of pancreatin and 1.75 g·L^−1^ of bile salt were added, and the mixture was shaken at 150 r·min^−1^ at 37 °C for 4 h. The solution and solid residue were collected in the same manner as in the gastric phase. The concentrations of heavy metals in the supernatant were analyzed by the atomic absorption spectrometer (Thermo Scientific iCE 3000 Series, Waltham, MA, USA) and atomic fluorescence spectrometer (Haiguang Instrument AFS-9710, Beijing, China). The total concentrations of heavy metals in the food samples were below the detection limits, and the variation of heavy metals concentration in the co-digestion system reflected the variation of heavy metals in the soil. All tests were performed in triplicate.

The heavy metal bioaccessibility for gastric and intestinal phases in vitro assay method was calculated as follows:

(1) $$BAC\;\left(\%\right)=\frac{C_{bioaccessible}}{C_{total}}\times 100$$

where $C_{bioaccessible}$ (mg·kg^−1^) was the heavy metal concentration in the supernatant obtained from gastrointestinal phase, $C_{total}$ (mg·kg^−1^) was the total concentration of soil heavy metals.

### 2.4. Human Health Risk Assessment

In order to quantify the risk of unintentional ingestion of contaminated soil particles combined with dietary, carcinogenic risk (CR) and hazard quotient (HQ) of children and adults were calculated based on the total and bioaccessible heavy metal concentration [24]. The specific calculation equations were detailed in the supporting materials. The cancer slope factors and reference dose value were used to evaluate heavy metal toxicity (Tables S1 and S2). Oral exposure was considered for potential health risk assessment in this study. The CR value between 1 × 10 ^−6^ and 1 × 10 ^−4^ generally considered acceptable, while CR > 1 × 10 ^−4^ indicate potential carcinogenic risk [25]. The HQ was applied to estimate human non-carcinogenic risk. There may be a potential non-carcinogenic risk when HQ exceeds 1 [26]. 

### 2.5. Data Analysis

The statistical analysis was performed using IBM SPSS 11.5 software. Pearson’s correlation was used for determining the dependence between variables (*p* < 0.05). Statistical significance (*p* < 0.05 was selected as a threshold) was tested with one-way ANOVA with Dunnett’s multiple comparisons test, or two-way ANOVA with Tukey’s multiple comparisons test as indicated in figure legends. All figures were visualized by Origin 2021.

## 3. Results

### 3.1. Soil Characteristics and Stabilization Effects

The basic physicochemical characteristics of tested soil were illustrated in Table 1. The soil was slightly alkaline with high nitrogen content. Soil As was 201.77 mg·kg^−1^, which was significantly higher than screen levels (20 mg∙kg^−1^) of environmental quality risk control standard for development land in China (GB36600–2018), while soil Zn was 1145.7 mg∙kg^−1^, indicating that soil was affected by anthropogenic activities. It worth noting that soil Fe content was 215.9 g∙kg^−1^, which might be associated with steel slag and ferroalloy likely used during the plant’s lifetime. After stabilized with 2% FeSO_4_, the bioavailable As concentration in treated soil decreased from 13.51 mg∙kg^−1^ to 11.15 mg∙kg^−1^ (*p* < 0.05), while the soil bioavailable Zn concentration increased from 15.70 mg∙kg^−1^ to 16.89 mg∙kg^−1^ (*p* < 0.05) (Table S3). It revealed that FeSO_4_ had an immobilization effect on As, but an opposite effect on Zn.

### 3.2. Effect of Dietary Components on Soil As Bioaccessibility

**Figure 1:**
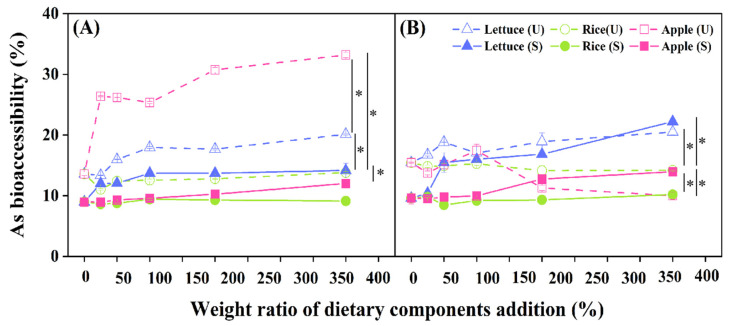
Bioaccessibility of As in the gastric (**A**) and intestinal (**B**) phase after addition of different dietary components before (U) and after (S) FeSO_4_ stabilization. Data are presented as the mean ± standard deviation (S.D.) of the three experimental groups. ANOVA analysis reveals the statistical differences between the stabilization treatments and dietary addition groups. An asterisk (*) indicates significant difference between the different dietary addition groups (*p* < 0.05). Summary of statistical information including *p* values is given in Table S4.

As bioaccessibility was significantly slightly higher in the intestinal phase than that in the gastric phase before FeSO_4_ stabilization without dietary addition (*p* < 0.05) (Figure 1), which was most likely attributed to digestive enzymes from pancreatic enzymes and bile involved in the breakdown of polysaccharides and proteins into smaller units in the intestinal environment [27]. After FeSO_4_ stabilization, As bioaccessibility in the gastric and intestinal phases were significantly decreased by 34.0% (from 13.57 mg∙kg^−1^ to 8.56 mg∙kg^−1^) (*p* < 0.05) and 37.9% (from 15.43 mg∙kg^−1^ to 8.59 mg∙kg^−1^) (*p* < 0.05), respectively, which revealed that FeSO_4_ was a good stabilizer for controlling health risk of soil As. 

The change of As bioaccessibility in the gastric and intestinal phases differed much with dietary. Before FeSO_4_ stabilization, the significant augmented effect of dietary components on As bioaccessibility in gastric phase was as follows: apple > lettuce > cooked rice (*p* < 0.05) (Figure 1A). Cooked rice had minimal effects on the soil As bioaccessibility, which indicated complex interaction between carbohydrate from cooked rice and soil As in gastrointestinal digestion. On one hand, carbohydrates dissolved into organic carbon could increase the release of As in the soil [28]; on the other hand, the migration of As might be limited by binding with hydrophobic micelles of carbohydrates [29]. The contribution of lettuce to bioaccessible As was limited, which might be due to dietary fiber in lettuce as the chelator for metals [30]. The augmented effect of apple on As bioaccessibility might be due to the large degradation of polyphenols in apple under acidic conditions (gastric acid) and the formation of other isomers, such as chlorogenic acid [31], which could promote the dissolution of As in the gastric phase. The results showed that dietary components affected soil As bioaccessibility in gastrointestinal solutions. Former studies reported As bioaccessibility differed much among various foods. For example, As bioaccessibility in quinoa was only approximately 40% because of rich minerals, vitamins and protein [32]. As bioaccessibility in boiled perch was reduced due to the release of As into boiling water and protein denaturation [33]. The food matrix played an important role in regulating the bioaccessibility of heavy metals because of the adsorption, chelation or reduction reaction between soluble metals and other food substrates (such as fiber, protein, macromolecules) after the gastrointestinal digestion process. 

In the co-digestion system of food and contaminated soil, there were significant differences in As bioaccessibility between gastric and intestinal phases (*p* < 0.05). Before stabilization with FeSO_4_, As bioaccessibility in gastric phase increased from 13.6% to 33.2% (*p* < 0.05) with increasing proportion of apple in the contaminated soil, while that in the intestinal phase decreased from 15.4% to 10.0% (*p* < 0.05). Notably, when apple proportion was 400% (apple/contaminated soil, *w/w*) in the digestive system, As bioaccessibility in gastric phase significantly increased by 114.6% (*p* < 0.05), which might be related to the polyphenols, such as anthocyanins, in apple. These polyphenols were unstable in the weakly alkaline intestinal environment [33], resulting in the relative smaller effects on As bioaccessibility in intestinal phase. After stabilized by FeSO_4_, soil As bioaccessibility in intestinal phase significantly increased from 9.59% to 22.21% with lettuce co-digested (*p* < 0.05) (Figure 1B). The enhancement effect of 400% apple on As bioaccessibility in the gastric phase significantly decreased from 114.6% to 33.8% (*p* < 0.05). Co-digested with a proportion of apple less than 100% in the gastric and intestine phases in soil stabilized by FeSO_4_ had no significant effect on As bioaccessibility (*p* > 0.05). The presence of FeSO_4_ reduced the promoting effect of apples on As bioaccessibility, indicating that FeSO_4_ not only controlled the health risk of As in soil but also reduced the promoting effect of dietary components on soil As bioaccessibility. 

### 3.3. Effect of Dietary Components on Soil Zn Bioaccessibility

**Figure 2:**
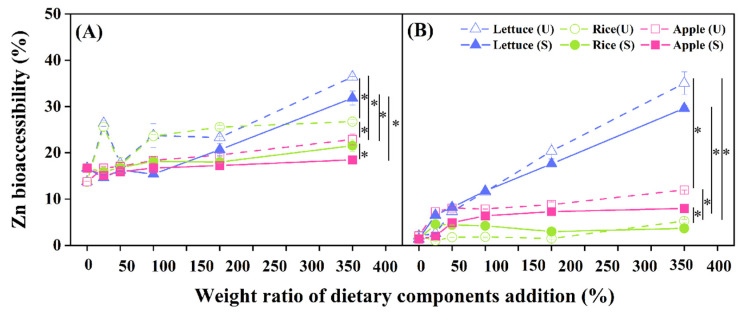
Bioaccessibility of Zn in the gastric (**A**) and intestinal (**B**) phases after addition of different dietary components before (U) and after (S) FeSO_4_ stabilization. Data are presented as the mean ± standard deviation (S.D.) of the three experimental groups. ANOVA analysis reveals the statistical differences between the stabilization treatments and dietary addition groups. An asterisk (*) indicates significant difference between the different dietary addition groups (*p* < 0.05). Summary of statistical information including *p* values is given in Table S4.

The effect of dietary components on soil Zn bioaccessibility in the gastric and intestinal phases before and after stabilization was shown in Figure 2. Soil Zn bioaccessibility in gastric phase were significantly higher than that in intestinal phase (*p* < 0.05). The low pH value (pH = 2.0 ± 0.2) in gastric phase might lead to high Zn solubility. Various enzymes in intestinal phase would undergo chemical reactions with Zn, such as adsorption, precipitation and complexation reactions, resulting in further reduction of bioaccessibility [34,35]. After FeSO_4_ stabilization, soil bioaccessible Zn increased significantly from 157.53 mg∙kg^−1^ to 208.48 mg∙kg^−1^ in the gastric phase (Figure 2A) (*p* < 0.05). Since the addition of FeSO_4_ made soil acidify, soil Zn was more easily activated as a typical cationic metal than As, without any secondary adsorption on the surface of Fe(III) hydroxides [36]. The ability of Zn to be weakly retained by the Fe(III) hydroxides at soil pH below the initial value of 8.29 could explain the negative effect of FeSO_4_ addition on Zn stability [14]. The bioaccessibility of Zn and As was significantly different before and after FeSO_4_ stabilization (*p* < 0.05), indicating that the geochemical properties of the two heavy metals in soil were so disparate, and thus resulted in different hazards to organisms. While FeSO_4_ could reduce Zn bioaccessibility in dietary-soil co-digestion system, and it was speculated that some dietary components enhance the adsorption capacity of Fe(III) hydroxides to Zn.

Dietary components could significantly increase Zn bioaccessibility both in gastric and intestinal phase before and after FeSO_4_ stabilization (*p* < 0.05). Interestingly, the effect of dietary components on As bioaccessibility was mainly in gastric phase, while the effect on Zn bioaccessibility was mainly in intestinal phase. For the intestinal phase (Figure 2B), the significantly promoted effects of dietary on Zn bioaccessibility were as follows: lettuce > apple > cooked rice (*p* < 0.05). The bioaccessible Zn in the gastric phase tended to increase linearly with cooked rice or apple dose after FeSO_4_ stabilization. A large amount of Zn bound to soil particles was released into the digestive juice and shifted to free state, resulting a tendency for increased Zn bioaccessibility in vitro models [37]. Overall, in the co-digestion system of dietary components and Zn contaminated soil, there was no regular difference in the effect of dietary components on Zn bioaccessibility in the gastric phase, but lettuce and apple improved Zn bioaccessibility in the intestinal phase. Zn bioaccessibility in intestinal phase had a linear increase trend with the increase of lettuce content in contaminated soil, and it significantly increased by 131.7% as lettuce proportion was 400% (lettuce/contaminated, soil *w/w*) (*p* < 0.05) in digestive system. The similar result was discovered by Smita et al. that amaranth increased Zn bioaccessibility from 89.7% to 124%, which might be due to the effects of β-carotene in vegetables [38]. 

### 3.4. As Speciation and Distribution

**Figure 3:**
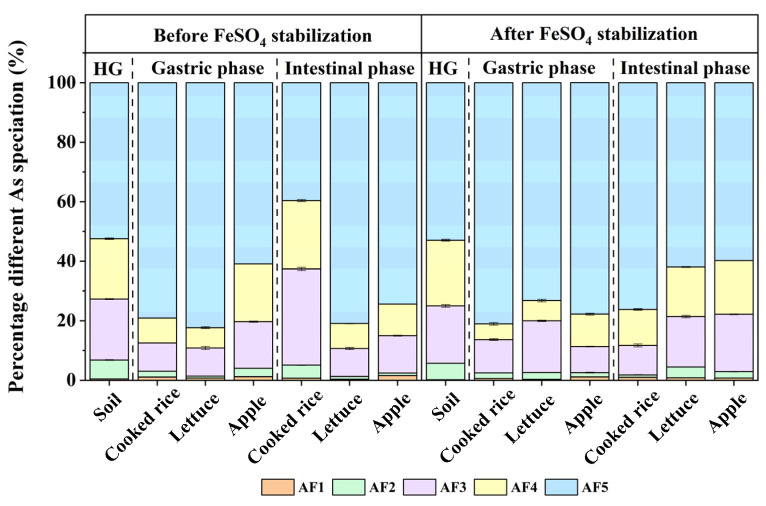
Transformation of As in the gastric and intestinal phases after addition of different dietary components before and after FeSO_4_ stabilization. Data are presented as the mean ± standard deviation (S.D.) of the three experimental groups. ANOVA analysis reveals the statistical differences between the stabilization treatments and dietary addition groups of different As fractions. Summary of statistical information including *p* values is given in Table S5. HG: original soil, AF1: non–specifically bound As, AF2: specifically bound As, AF3: amorphous hydrous oxide bound As, AF4: crystalline hydrous oxide bound As, AF5: residual As.

As bioaccessibility was less than 35% in digestive juices based on PBET, which showed that soil As could not be completely dissolved from the soil matrix. To further understand the potential mechanisms of chemical stabilization on As bioaccessibility, the speciation of As in stimulative co-digestive residues was studied by the sequential extraction procedure (Figure 3). Before FeSO_4_ stabilization, AF1 (non–specifically bound As), AF2 (specifically bound As), AF3 (amorphous hydrous oxide bound As), AF4 (crystalline hydrous oxide bound As) and AF5 (residual As) in the initial contaminated soil accounted for 0.5%, 6.3%, 20.5%, 20.2% and 52.5%, respectively. The results showed that most of AF1, AF2, and AF3 were bioaccessible. The low proportion of AF1 and AF2 factions in the soil might be due to natural weathering effects, and these easily leached As could be largely reduced by storm water runoff or infiltration or transformed into other more stable fractions [39]. The residual As was mainly composed of oxyanions, which were tightly bound to the mineral components in soil (such as arsenopyrite (FeAsS), coniform (CaCu(AsO_4_)OH)), which were more insoluble than other forms of As [40,41]. After FeSO_4_ stabilization, the proportions of AF1, AF2 and AF3 decreased to 0.2%, 5.5% and 19.3%, while AF4 and AF5 increased to 22.1% and 53.0%, illustrating that FeSO_4_ could effectively stabilize As. The Fe(OH)_3_ obtained by the reaction provided abundant adsorption site for As [42]. Thus, FeSO_4_ could convert the easily available As fractions into highly stable fractions.

Dietary components had a significant effect on As fractions in co-digestion system of food and As contaminated soil (Figure 3) (*p* < 0.05). AF1 was defined as an outer-sphere, which represented prevailing As absorbed on the surface of soil mineral by electrostatic attraction, and AF2 represents inter-sphere As complexes with coordination-covalent bonding [43]. AF1 and AF2 could be considered to be the two most unstable components in the soil and the main sources of bioaccessible As. Non-specifically and specifically adsorbed As were considered soluble in digestive juices [44]. The addition of 400% dietary components (especially lettuce) reduced the proportion of AF1 + AF2 in the residue, due to the conversion of most of the non-specifically and the specifically bound fractions to bioaccessible As in digestive juice, indicating that dietary could promote the release of the more active fractions of As in the soil in the gastric and intestinal phases. Cooked rice and lettuce affected the distribution of As fractions in the residue after gastric phase by increasing the proportion of residual As by 79.08% and 82.33% (*p* < 0.05), respectively. Before FeSO_4_ stabilization, the apple had a limited effect on the enhancement of AF5, significantly increasing by 16% (*p* < 0.05) in co-digestion residue in the gastric phase compared to initial contaminated soil residues. In addition, AF4 and AF5 in the residue was almost not affected by apples, indicating that the increase in the bioaccessible As in apples might be mainly due to the promotion of soil non-specifically and the specifically bound As. 

**Figure 4:**
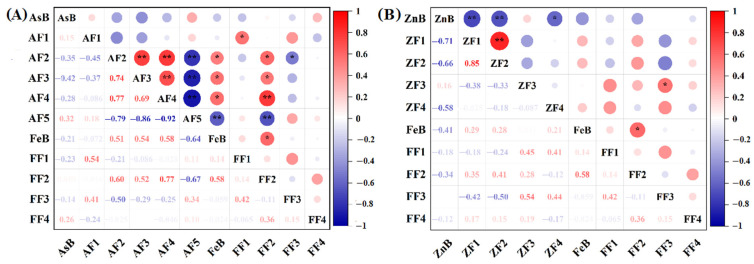
The correlation coefficients of different species of As (**A**) and Zn (**B**) with Fe fractions in soils. AsB: As bioaccessibility, AF1: non–specifically bound As, AF2: specifically bound As, AF3: amorphous hydrous oxide bound As, AF4: crystalline hydrous oxide bound As, AF5: residual As, ZnB: Zn bioaccessibility, ZF1: acid-exchangeable Zn, ZF2: reducible Zn, ZF3: oxidizable Zn, ZF4: residual Zn, FeB: Fe bioaccessibility, FF1: acid-exchangeable Fe, FF2: reducible Fe, FF3: oxidizable Fe, FF4: residual Fe, * *p* < 0.05, ** *p* < 0.01.

Studies have shown that the As bioaccessibility was related to the As fractions [45]. However, none of the As fractions was significantly associated with the As bioaccessibility in the gastric and intestinal phases (Figure 4). The As bioaccessibility might be simultaneously affected by other factors. Soil Fe was able to describe 96% of the changes in As bioaccessibility in different type of soils [46]. Although there was no correlation between Fe bioaccessibility and As bioaccessibility, FF2 fraction of Fe was positively correlated with AF2, AF3 and AF4 but negatively correlated with AF5 (Figure 4). FF2 was the most important Fe oxide minerals associated with As. The good correlation between As fractions and partitioning of Fe oxide minerals has been found in previous studies [47]. Fe(III) oxide minerals in the residue were more stable and had better crystallinity [48]. In this study, the dominant As fractions were strongly associated with the stable speciation of Fe oxide minerals in contaminated soils. Accordingly, the transformation of Fe fractions could affect the distribution of As fractions in soils. 

### 3.5. Zn Speciation and Distribution

**Figure 5:**
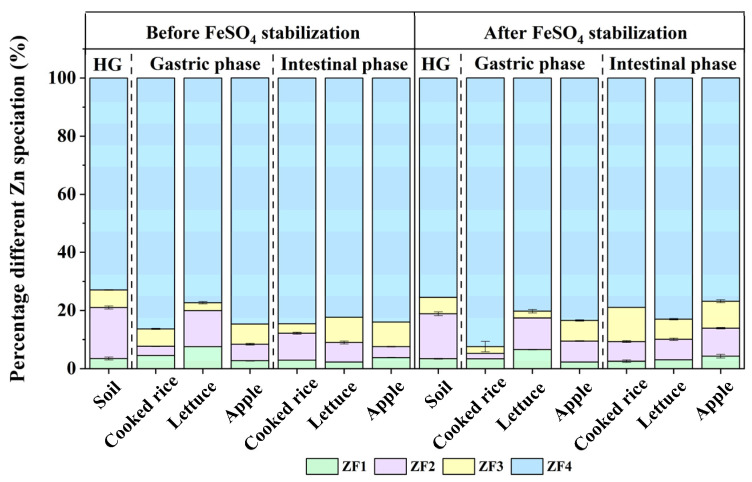
Transformation of Zn in the gastric and intestinal phases after addition of different dietary components before and after FeSO_4_ stabilization. Data are presented as the mean ± standard deviation (S.D.) of the three experimental groups. ANOVA analysis reveals the statistical differences between the stabilization treatments and dietary addition groups of different Zn fractions. Summary of statistical information including *p* values is given in Table S5. HG: original soil, ZF1: acid-exchangeable Zn, ZF2: reducible Zn, ZF3: oxidizable Zn, ZF4: residual Zn.

The residual Zn in co-digestive residues before and after FeSO_4_ stabilization were 72.9% and 75.5% (*p* < 0.05), respectively, indicating that FeSO_4_ had limited effects on the stability of Zn (Figure 5). Meanwhile, after extraction by the gastric and intestinal phases, the proportion of ZF4 (residual fraction) and ZF3 (oxidizable fraction) decreased, while the proportion of ZF1 (acid-exchangeable fraction) and ZF2 (reducible fraction) increased. The complex chemical environment of gastric and intestinal phases might increase the mobility of Zn. In the co-digestion system of food and contaminated soil, the distribution of Zn was also dominated by residual fractions (Figure 5). ZF1 and ZF2 had been proved to have high mobility and easily migrated to the organisms from the soil solid [5]. In this study, the proportion of ZF1 + ZF2 was reduced compared to the initial contaminated soil. It was for two reasons that Zn in the fraction of bioaccessibility in digestive juice: the release of the acid-exchangeable fraction of Zn that was loosely attached to the residue under dietary addition, and the residual fraction of Zn was easily activated under reduction conditions following the disintegration of the oxides or hydroxides [5]. Before FeSO_4_ stabilization, the effect of cooked rice and apple had similar effects on Zn bioaccessibility in the gastric and intestinal phases. The residue Zn content changed less, but the oxidizable fraction and acid-exchangeable fraction were converted to the free phase, which was consistent with the conclusion that cooked rice and apples had a smaller effect on Zn bioaccessibility relative to lettuce obtained above. The Zn bioaccessibility in lettuce was higher than that of cooked rice and apples. In addition, some variable related to the chemical fractions of the metal estimated by the BCR method were correlated with most of the bioaccessibility of metals [39]. The ZF1, ZF2 and ZF4 fractions of Zn were significantly negatively correlated with the Zn bioaccessibility (*p* < 0.01) (Figure 4), indicating that the Zn bioaccessibility could be formed in a short period of time through the transformation of more endogenous morphological components, such as acid-exchangeable Zn, reducible Zn and residual Zn. 

### 3.6. Human Health Risk of Soil Heavy Metals

**Figure 6:**
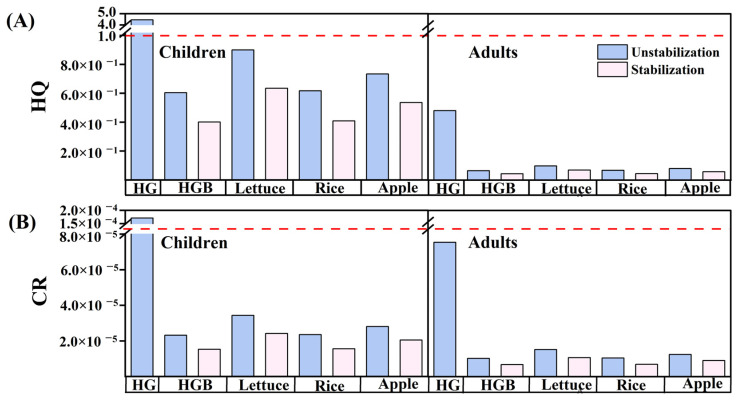
Hazard quotient (HQ) (**A**) and carcinogenic risk (CR) (**B**) of dietary components before and after FeSO_4_ stabilization. HG: risk assessment values based on the total concentration of soil heavy metals. HGB: risk assessment values based on the bioaccessibility of soil heavy metals.

The carcinogenic and non-carcinogenic risk assessments for adults and children based on the ingestion of heavy metal contaminated soil in gastric and intestinal phases were shown in Figure 6. Children were more vulnerable to heavy metals due to their physiological characteristics and living habits [25]. In the experimental group without addition of dietary components, it was evident that direct ingestion posed the greatest risk to human health in the soil before stabilization. Among them, the carcinogenic risk (CR = 1.71 × 10 ^−4^) and non-carcinogenic risk (HQ = 4.45) in children exceed the critical value. However, the HQ and CR values changed significantly, which decreased by 33.97% and 33.59% (*p* < 0.05) after FeSO_4_ stabilization. Although HQ values was less than the safety threshold 1, the CR values was between 1 × 10 ^−6^ and 1 × 10 ^−4^. It indicated that the addition of FeSO_4_ could reduce the impact of human ingestion of heavy metals in contaminated soil on the human non-carcinogenic risk, but there might still be carcinogenic risks. Additionally, the health risk values assessed based on the bioaccessibility in the gastric phase was significantly reduced.

Treatments with the maximum amount (400%, *w*/*w*) of dietary components had the highest bioaccessibility of heavy metals. The order of the HQ and CR values among dietary components was: lettuce > apple > cooked rice (*p* < 0.05). It should be noted that lettuce and apples might increase the human health risk. Especially for children, lettuce increased the HQ value of the gastric phase from 0.60 to 0.90, increasing the risk by 49%. Although the intake of lettuce and apples also increased the CR value, the lower carcinogenic risk value was negligible. The intake of cooked rice had no obvious effect on the risk to human health in the gastric phase, indicating that cooked rice could not regulate the exposure of metals by reducing the As and Zn bioaccessibility. In addition, the contribution of Zn to non-carcinogenic risk was negligible (Figure S1). As was the main source of potential non-carcinogenic risk caused by soil ingestion before and after FeSO_4_ stabilization, accounting for 98.56~99.44% and 96.7~98.69% of the HQ of children and adults, respectively. It was noting that all evaluations of As were based on elemental As, but As existed in different fractions in the gastrointestinal environment, which posed different toxicity to human. Therefore, it was necessary to integrate different fractions of As to assess the impact on human health. In addition, a single route of metal exposure was not enough to pose a significant health risk to human. Multiple routes should not be ignored, such as food ingestion and the combined effects of metal in human. Environmental health risk assessment should pay more attention to the study of food types and ingestion dosages. Based on the health risk assessment results of this study, it was recommended to adjust the dietary structures, especially the intake of lettuce and apples should be controlled.

## 4. Conclusions

In this study, FeSO_4_ was a good stabilizer for controlling the exposure risk of As in soil. Before FeSO_4_ stabilization, the augmented effects of dietary components on the As bioaccessibility in gastric phase were as follows: apple > lettuce > cooked rice (*p* < 0.05). Dietary components had various augmented effects on the bioaccessibility of As and Zn in the gastric and intestinal phases. The augmented effect was mainly due to the conversion of the non-specifically and specifically bound heavy metals in soils and generally increased with the addition proportion. Cooked rice and lettuce could redistribute the As fraction in the co-digestive residues. However, the effect of dietary components on Zn bioaccessibility in the gastric phase had no regular differences, but an increasing trend of Zn bioaccessibility was found positively correlated with the lettuce dosage in the intestinal phase. Zn bioaccessibility could be formed through the transformation of more endogenous morphological components. The application of stable materials and the intake of dietary component changed the human health risks of heavy metals. The addition of FeSO_4_ could reduce the effect of human intake of heavy metals in contaminated soil on human non-carcinogenic risk, but there might still be carcinogenic risk. Among them, cooked rice did not regulate the exposure of dietary component by reducing the bioaccessibility of As and Zn. Therefore, more attention should be paid to the combined effect of dietary types and intake dose when evaluating the human health risks of contaminated soils.

## Figures and Tables

**Table 1:** Physicochemical properties of the experimental soil.

Physicochemical Properties	Value ± SD
pH	8.20 ± 0.01
CEC (cmol∙kg^−1^)	11.30 ± 0.2
SOM (g∙kg^−1^)	18.70 ± 0.6
TP (%)	0.16 ± 0.00
TN (mg∙kg^−1^)	108.00 ± 2.5
TK (mg∙kg^−1^)	1.14 ± 0.01
Fe_2_O_3_ (%)	30.84 ± 1.44
Tas (mg∙kg^−1^)	201.77 ± 2.56
TZn (mg∙kg^−1^)	1145.71 ± 13.42

CEC: cation exchange capacity; SOM: soil organic matter; TP: total phosphorus content; TN: total nitrogen content; TK: total potassium content; Tas: total As content; TZn: total Zn content; SD: standard deviation.

## Data Availability

Data are available from the corresponding author upon request.

## Supplementary Materials

The following supporting information can be downloaded at: https://www.mdpi.com/article/10.3390/toxics11010023/s1, Figure S1: As and Zn accounted for HQ values in adults and children in the co-digestion system of food and contaminated soil; Table S1: Calculation parameters and values used in health risk assessment model to evaluate exposure risks of soil; Table S2: Corresponding reference dose (*RfD*) and slope factors (*CSF*) values of metals in soil [49,50,51]; Table S3: Bioavailability concentration of metals in experimental soil before and after FeSO_4_ stabilization. Table S4: Figure 1 and Figure 2 Statistical Analyses; Table S5: Figure 3 and Figure 5 Statistical Analyses.
