# Supplementary material for: In Vitro Bioaccessibility and Health Risk Assessment of Arsenic and Zinc Contaminated Soil Stabilized by Ferrous Sulfate: Effect of Different Dietary Components
Source: Toxics. 2022 Dec 26;11(1):23. doi: 10.3390/toxics11010023 (PMC9863096; doi:10.3390/toxics11010023)
Supplement: Supplementary file 1 [file toxics-11-00023-s001.zip › toxics-2090625 Supplementary.pdf]

toxics

MDPI

Supplementary Materials: In Vitro Bioaccessibility and Health Risk Assessment of Arsenic and Zinc Contaminated Soil Stabilized by Ferrous Sulfate: Effect of Different Dietary Components

Yi Fang, Yuxue Cui, Xiaoli Mou, Li Lu, Jiali Shentu and Min Zhu

Human health risk assessment

Average daily intake (ADD) of soil heavy metal accidentally ingested was calculated as follows:

$$ADD = \frac{C_{total} \times I_R \times E_F \times E_D \times 10^{-6}}{BW \times AT} \quad (1)$$

where C_{total}(mg kg^{-1}) was the total concentration of soil heavy metals, and the remaining parameters (I_R, E_F, E_D, BW and AT) were shown in Table S1.

The HQ and CR based on total heavy metals concentration and heavy metal bioaccessibility were estimated as follows:

$$HQ_{total} = \frac{ADD}{RfD} \quad (2)$$

$$HQ_{bioaccessible} = \frac{ADD \times BAC}{RfD} \quad (3)$$

$$CR_{total} = ADD \times CSF \quad (4)$$

$$CR_{bioaccessible} = ADD \times BAC \times CSF \quad (5)$$

where BAC refers to the heavy metal bioaccessibility. The specific values of oral reference dose (RfD) and cancer slope factors (CSF) were shown in Table S2.

Table S1. Calculation parameters and values used in health risk assessment model to evaluate exposure risks of soil.

Parameter	Description	Unit	Children	Adult	Reference
I_R	Ingestion rate of soil	mg day^{-1}	200	100	
E_F	Exposure frequency	day year^{-1}	350	350	[1,2]
E_D	Exposure duration	years	6	24	
BW	Average body weight	kg	15	70	
AT	Average time of exposure to contaminated soils	day	365 × E_D (non-carcinogenic) 365 × 70 (carcinogenic)		

Table S2. Corresponding reference dose (*RfD*) and slope factors (*CSF*) values of metals in soil.

Elements	***RfD*** **(mg (kg d)$^{-1}$) [2,3]**	***SF*** **((kg d) mg^{-1}) [1]**
Pb	3.50E-03	8.50E-03
Ni	2.00E-02	n/a
Cu	4.00E-02	n/a
Zn	3.00E-01	n/a
Cd	1.00E-03	6.1E+00
Cr	3.00E-03	8.50E-03

Note: n/a not available.

Table S3. Bioavailability concentration of metals in experimental soil before and after $FeSO_4$ stabilization.

Bioavailability	Before $FeSO_4$ stabilization	After $FeSO_4$ stabilization	*p* value
As (mg kg^{-1})	13.51	11.15	**
Zn (mg kg^{-1})	15.70	16.89	**
p value	**	**	

Note: ** means $p < 0.01$.

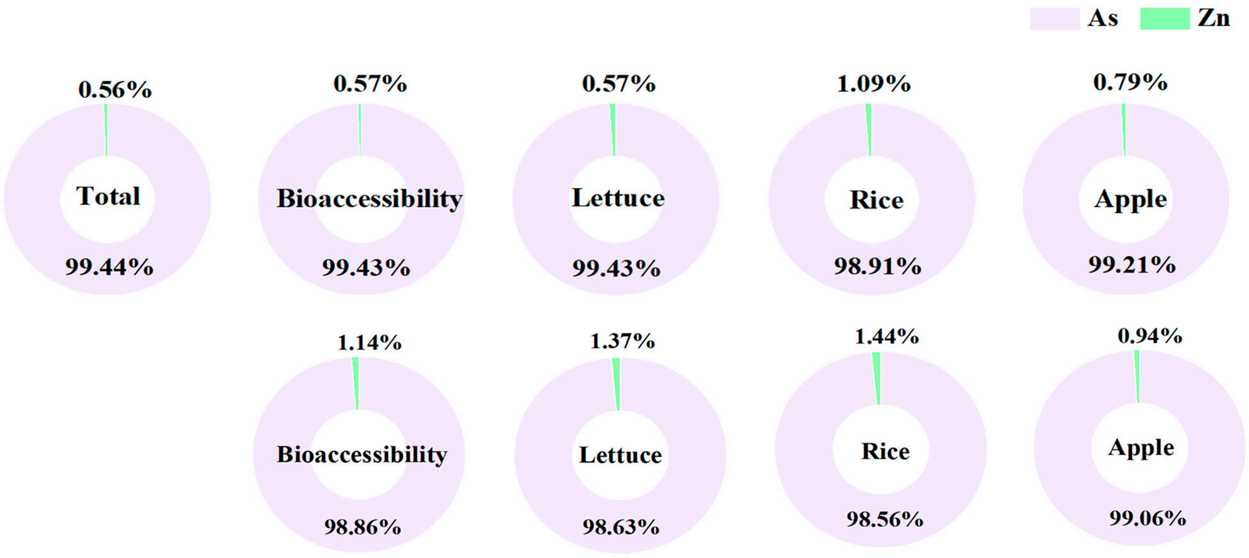

Figure S1. As and Zn accounted for HQ values in adults and children in the co-digestion system of food and contaminated soil.

References

1. Huang, J.; Wu, Y.; Sun, J.; Li, X.; Geng, X.; Zhao, M.; Sun, T.; Fan, Z. Health risk assessment of heavy metal(loid)s in park soils of the largest megacity in China by using Monte Carlo simulation coupled with Positive matrix factorization model. *J. Hazard. Mater*. **2021**, *415*, 125629.
2. Sun, J.; Zhao, M.; Huang, J.; Liu, Y.; Wu, Y.; Cai, B.; Han, Z.; Huang, H.; Fan, Z. Determination of priority control factors for the management of soil trace metal(loid)s based on source-oriented health risk assessment. *J. Hazard. Mater*. **2022**, *423*, 127116.
3. Men, C.; Liu, R.; Wang, Q.; Miao, Y.; Wang, Y.; Jiao, L.; Li, L.; Cao, L.; Shen, Z.; Li, Y.; et al. Spatial-temporal characteristics, source-specific variation and uncertainty analysis of health risks associated with heavy metals in road dust in Beijing, China. *Environ. Pollut*. **2021**, *278*, 116866.
